# Supplementary material for: Accuracy of Machine Learning Algorithms for the Classification of Molecular Features of Gliomas on MRI: A Systematic Literature Review and Meta-Analysis
Source: Cancers (Basel). 2021 May 26;13(11):2606. doi: 10.3390/cancers13112606 (PMC8198025; doi:10.3390/cancers13112606)
Supplement: Supplementary file 1 [file cancers-13-02606-s001.zip › cancers-1193540-supplementary.pdf]

Supplementary Materials
The search strategy for a) Medline (accessed through PubMed), b) EMBASE, c) The Cochrane Library is displayed here.

a) Medline

1. (360 hits)

(((("Glioma"[Mesh] OR "Brain Neoplasms"[Mesh:NoExp] OR Glioma*[tiab] OR brain neoplasm*[tiab] OR Glioblastoma*[tiab] OR Glial Cell Tumo*[tiab]) AND (("Artificial Intelligence"[Mesh:NoExp] OR "Machine Learning"[Mesh] OR "Neural Networks, Computer"[Mesh] OR "Neuroimaging/classification"[Mesh] OR Advanced neuroimaging[tiab] OR AI[tiab] OR artificial intelligence[tiab] OR deep learning[tiab] OR machine learning[tiab] OR neural network*[tiab] OR Radiogenomic*[tiab] OR Radiomic[tiab] OR Radiomics[tiab]))) AND (("Area Under Curve"[Mesh] OR "Sensitivity and Specificity"[Mesh:NoExp] OR AUC[tiab] OR Area under curve[tiab] OR Area under the curve[tiab] OR area under the receiver operator curve[tiab] OR accuracy[tiab]))) AND (magnetic resonance imaging[MeSH Terms] OR MRI[tiab] OR magnetic resonance imaging[tiab])

2. (224 hits)

(((machine learning[Title/Abstract]) OR (((((((((ai artificial intelligence[MeSH Terms]) OR ais artificial intelligence[MeSH Terms]) OR model, neural network[MeSH Terms]) OR models, neural network[MeSH Terms]) OR neural network model[MeSH Terms]) OR neural network models[MeSH Terms]) OR neural networks computer[MeSH Terms]) OR neural network computer[MeSH Terms])) OR (((deep learning[Title/Abstract]) OR artificial intelligence[Title/Abstract]) OR neural network model[Title/Abstract])))) AND (((((brain neoplasm[MeSH Terms]) OR brain tumor[MeSH Terms]) OR brain cancer[MeSH Terms])) AND ((((brain neoplasm[Title/Abstract]) OR brain tumor[Title/Abstract]) OR brain cancer[Title/Abstract]) OR neuro-oncology))

3. (24 hits)

((((((((“Glioma/diagnostic imaging”[Mesh]) OR “Brain Stem Neoplasms/diagnostic imaging”[Mesh]) OR “Central Nervous System Neoplasms/diagnostic imaging”[Mesh]) OR “Brain Neoplasms/diagnostic imaging”[Mesh]) OR “Nervous System Neoplasms/diagnostic imaging”[Mesh]) AND ((((“Artificial Intelligence”[Mesh]) OR “Neural Networks, Computer”[Mesh]) OR “Machine Learning”[Mesh]) OR “Deep Learning”[Mesh])) AND ((“Brain Neoplasms/diagnostic imaging”[Mesh]) AND “Diagnostic Imaging”[Mesh])) AND ((“Magnetic Resonance Imaging”[Mesh]) AND “Brain Neoplasms”[Mesh])) AND ((radiomics) OR (radiogenomics))

4. (17 hits)

((neuro-oncology) AND (glioma classification)) AND (((((neural networks[Title/Abstract]) OR (machine learning[Title/Abstract])) OR (deep learning[Title/Abstract])) OR (artificial intelligence[Title/Abstract])) OR ((((("Artificial Intelligence"[Mesh]) OR "Machine Learning"[Mesh]) OR "Deep Learning"[Mesh]) OR "Neural Networks, Computer"[Mesh]) OR "Neuroimaging/classification"[Mesh]))

5. (15 hits)

(((neuroradiology) AND (glioma)) AND (classification[Title/Abstract])) AND (((((neural networks[Title/Abstract]) OR (machine learning[Title/Abstract])) OR (deep learning[Title/Abstract])) OR (artificial intelligence[Title/Abstract])) OR ((((("Artificial Intelligence"[Mesh]) OR "Machine Learning"[Mesh]) OR "Deep Learning"[Mesh]) OR "Neural Networks, Computer"[Mesh]) OR "Neuroimaging/classification"[Mesh]))

6. (28 hits)

(((artificial intelligence[Title/Abstract]) OR (neural networks[Title/Abstract])) AND (glioma[Title/Abstract])) AND (((AUC) OR (area under the recieve operator curve)) OR (accuracy))

7. (13 hits)

((((glioma[Title/Abstract]) OR (glioma[MeSH Terms])) AND ((((artificial intelligence[Title/Abstract]) OR (neural networks[Title/Abstract])) OR (neural network[Title/Abstract])) OR (machine learning[Title/Abstract]))) AND (((area under curve[MeSH Terms]) OR (area under receiver operator curve[Title/Abstract])) OR (AUC[Title/Abstract]))) AND ((((neuro-oncology) OR (neuro-radiology)) OR (neuroimaging)) OR (advanced neuroimaging))

8. (28 hits)

((((((((((ai artificial intelligence[MeSH Terms]) OR ais artificial intelligence[MeSH Terms]) OR model, neural network[MeSH Terms]) OR models, neural network[MeSH Terms]) OR neural network model[MeSH Terms]) OR neural network models[MeSH Terms]) OR neural networks computer[MeSH Terms]) OR neural network computer[MeSH Terms])) AND (((deep learning[Title/Abstract]) OR artificial intelligence[Title/Abstract]) OR neural network model[Title/Abstract])) AND ((((brain neoplasm[Title/Abstract]) OR brain tumor[Title/Abstract]) OR brain cancer[Title/Abstract]) OR neuro-oncology)) AND (((brain neoplasm[MeSH Terms]) OR brain tumor[MeSH Terms]) OR brain cancer[MeSH Terms])

9. (7 hits)

((((genetics[Title/Abstract]) OR genetics[MeSH Terms])) AND ((((((((((ai artificial intelligence[MeSH Terms]) OR ais artificial intelligence[MeSH Terms]) OR model, neural network[MeSH Terms]) OR models, neural network[MeSH Terms]) OR neural network model[MeSH Terms]) OR neural network models[MeSH Terms]) OR neural networks computer[MeSH Terms]) OR neural network computer[MeSH Terms])) OR (((deep learning[Title/Abstract]) OR artificial intelligence[Title/Abstract]) OR neural network model[Title/Abstract]))) AND (((((brain neoplasm[MeSH Terms]) OR brain tumor[MeSH Terms]) OR brain cancer[MeSH Terms])) AND ((((brain neoplasm[Title/Abstract]) OR brain tumor[Title/Abstract]) OR brain cancer[Title/Abstract]) OR neuro-oncology))

10. (9 hits)

(((((((((((ai artificial intelligence[MeSH Terms]) OR ais artificial intelligence[MeSH Terms]) OR model, neural network[MeSH Terms]) OR models, neural network[MeSH Terms]) OR neural network model[MeSH Terms]) OR neural network models[MeSH Terms]) OR neural networks computer[MeSH Terms]) OR neural network computer[MeSH Terms])) OR (((deep learning[Title/Abstract]) OR artificial intelligence[Title/Abstract]) OR neural network model[Title/Abstract]))) AND ((genetics[Title/Abstract]) OR genetics[MeSH Terms])) AND radiomics[Title/Abstract]

b) EMBASE

1. (369 hits)

(glioma/ or glioma*.ti,ab,kw. or glioblastoma*.ti,ab,kw. or glial cell tumo*.ti,ab,kw.) and (artificial intelligence/ or exp machine learning/ or artificial neural network/ or advanced neuroimaging.ti,ab,kw. or AI.ti,ab,kw. or artificial intelligence.ti,ab,kw. or deep learning.ti,ab,kw. or machine learning.ti,ab,kw. or neural network*.ti,ab,kw. or radiogenomic*.ti,ab,kw. or radiomic.ti,ab,kw. or radiomics.ti,ab,kw.) and (area under the curve/ or AUC.ti,ab,kw. or area under curve.ti,ab,kw. or area under the curve.ti,ab,kw. or area under the receiver operator curve.ti,ab,kw. or accuracy.ti,ab,kw.) and (exp nuclear magnetic resonance imaging/ or MRI.ti,ab,kw. or magnetic resonance imaging.ti,ab,kw.) (with limit: english language and last 5 years)

c) The Cochrane Library

1. (0 hits)

(glioma*:ti,ab,kw OR glioblastoma*:ti,ab,kw) AND ([mh artificial intelligence] OR [mh machine learning] OR [mh deep learning] OR ''advanced neuroimaging'':ti,ab,kw OR AI:ti,ab,kw OR ''artificial intelligence'':ti,ab,kw OR ''deep learning'':ti,ab,kw OR ''machine learning'':ti,ab,kw) AND

(AUC:ti,ab,kw OR ′′area under the curve′′:ti,ab,kw OR ′′area under the receiver operator curve′′:ti,ab,kw) AND (MRI:ti,ab,kw OR [mh magnetic resonance imaging])
